# Do we have the right models for scaling up health services to achieve the Millennium Development Goals?

**DOI:** 10.1186/1472-6963-11-336

**Published:** 2011-12-14

**Authors:** Savitha Subramanian, Joseph Naimoli, Toru Matsubayashi, David H Peters

**Affiliations:** 1 Johns Hopkins University Bloomberg School of Public Health, Department of International Health, 615 N. Wolfe St., Baltimore, MD, 21205, USA; 2 U.S. Agency for International Development GH/AA, Rm 5.07-101 RRB 1300 Pennsylvania Ave., NW Washington, DC, 20523, USA

## Abstract

**Background:**

There is widespread agreement on the need for scaling up in the health sector to achieve the Millennium Development Goals (MDGs). But many countries are not on track to reach the MDG targets. The dominant approach used by global health initiatives promotes uniform interventions and targets, assuming that specific technical interventions tested in one country can be replicated across countries to rapidly expand coverage. Yet countries scale up health services and progress against the MDGs at very different rates. Global health initiatives need to take advantage of what has been learned about scaling up.

**Methods:**

A systematic literature review was conducted to identify conceptual models for scaling up health in developing countries, with the articles assessed according to the practical concerns of how to scale up, including the planning, monitoring and implementation approaches.

**Results:**

We identified six conceptual models for scaling up in health based on experience with expanding pilot projects and diffusion of innovations. They place importance on paying attention to enhancing organizational, functional, and political capabilities through experimentation and adaptation of strategies in addition to increasing the coverage and range of health services. These scaling up approaches focus on fostering sustainable institutions and the constructive engagement between end users and the provider and financing organizations.

**Conclusions:**

The current approaches to scaling up health services to reach the MDGs are overly simplistic and not working adequately. Rather than relying on blueprint planning and raising funds, an approach characteristic of current global health efforts, experience with alternative models suggests that more promising pathways involve "learning by doing" in ways that engage key stakeholders, uses data to address constraints, and incorporates results from pilot projects. Such approaches should be applied to current strategies to achieve the MDGs.

## Background

It is widely agreed that health services in developing countries need to be "scaled up" to achieve the Millennium Development Goals (MDGs). The MDGs, which were adopted in 2000 at the United Nations, set ambitious goals for reducing child and maternal mortality, combating HIV/AIDS and malaria, and achieving high levels of coverage for basic health services. New global health initiatives (such as the Global Fund to Fight AIDS, Tuberculosis, and Malaria (GFATM), the World Bank Multi-Country HIV/AID Program (MAP), the US President's Emergency Fund for AIDS Relief (PEFFAR), the GAVI Alliance, the Roll Back Malaria Partnership, and the Stop TB Partnership), and increased financial resources [1] have raised expectations to deliver health programs at large scale. Although not explicitly defined by the global health initiatives, a working definition of scaling up has been proposed as "an ambition or process of expanding the coverage of health interventions" [2].

Most of the recent emphasis on scaling up has focused on achieving high coverage rates of health services and reducing mortality, rather than the processes for how to scale up. The MDGs are identical for all countries, which in the case of childhood mortality, sets the target as a two-thirds reduction in child mortality rate between 1990 and 2015, the equivalent of an average annual decline of 4.3%. For the most part, the scaling up process is seen as the replication of specific health interventions (e.g. immunization, skilled birth attendance, integrated management of childhood illness, etc.) that have been shown to be cost-effective in a limited number of settings - usually a research setting or special projects conducted in a few countries. The intervention is expected to be delivered through a better-resourced and enlarged public health delivery system by replicating a similar package of interventions at more points of service delivery, often through model district health systems. HIV/AIDS programs are somewhat different in that they also enlist large numbers of non-governmental organizations (NGOs) for some of the interventions. The process of expanding coverage also involves the swift disbursement of funds, expanding partnerships, ensuring sustainable funding and promoting ownership, particularly at central levels [3-5]. These assumptions have been used to estimate the costs of scaling up of several health services intended to achieve the MDGs [6], and to estimate the human resources needed to provide them [7]. Although in some cases adjustments are made for expected economies of scale, the expanding coverage of specific health interventions is largely expected to be independent of each other.

**Figure 1:**
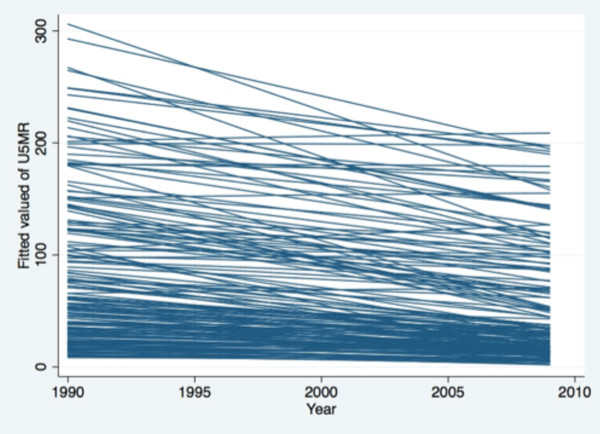
**Trends in Under-Five Mortality for all Low and Middle Income Countries, 1990-2009**. Note: Each line represents one country's trend, based on a multi-level model with random intercepts and random slopes for each country [10] Source: World Development Indicators [11].

**Figure 2:**
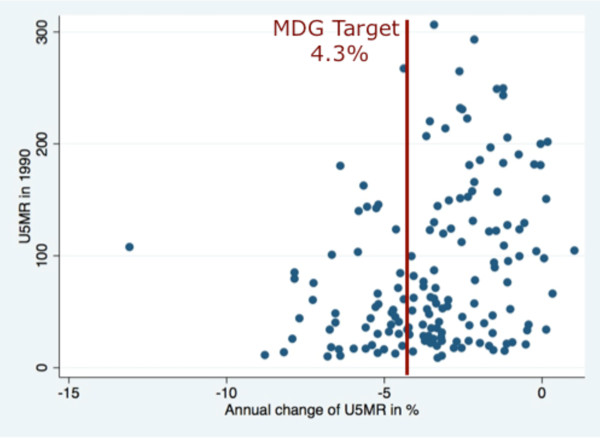
**Comparison of National Under Five Mortality Rates in 1990 and Subsequent Annual Rates of Change for Low and Middle Income Countries**. Note: Each dot represents one country, with the trend based on a multi-level model with random intercepts and random slopes for each country [10] Source: World Development Indicators [11].

Unfortunately, many countries are not on track to achieve the MDG health goals by the end date of 2015 [8], with one quarter to one half of all countries projected to not achieve their target levels for health services [9]. Previous analysis of national trends in coverage of MDG-related health services where there exists sufficient data for trend analysis demonstrated that countries have very different rates of change of coverage [9]. It was also found that changes in the rates of coverage of some health services are associated with the rates of change of other services (the services with sufficient data to assess annual rates of change include childhood immunization coverage, skilled birth attendance, tuberculosis treatment completion, and tuberculosis case detection). Using World Development Indicators data and the same multi-level statistical models as were used to assess changes in health services [9-11], we also analyzed the annual rates of change in under five mortality for each low and middle income country between 1990 to 2009 (Figure 1). Each line represents one country, and suggests that rates of change are highly divergent from one country to the next, and that the concept of an average country or average rate of change does not represent past experience. Figure 2 uses the same data to plot a country's under five mortality rate in 1990 against its subsequent annual rate of change, demonstrating that many countries are not reaching the target level of 4.3% reduction per year, and that there are very few clusters of countries with similar rates of change and starting points. These results suggest that even if common sets of health interventions and common health goals are being pursued by the MDGs, the rates at which they are being scaled up is quite different, and with some countries even losing coverage and increasing mortality.

Notably absent from much of the discussion around scaling up to reach MDG targets has been the logic models or theories of change that can guide practice and research [12], though a number of recent papers have examined why scaling up is not happening at the targeted rates. A recent review of the literature on scaling up in international health identified a number of common constraining factors, including the lack of absorptive capacity, weak health systems, human resource limitations, and high costs [13]. On the other hand, strong leadership and management, realistic financing, and technical innovation are believed to be common characteristics of successful large scale health programs [13,14]. However, there has been little attention paid to understanding why these factors occur and how to change them in ways that would guide practice for scaling up [12]. Recent publications that have addressed how scaling up occurs have concluded that scaling up processes are complex [15], with changes in political context and program management factors being major sources of variation in how scaling up occurs [16,17]. There have also been warnings against the over-reliance on "gold standard" evidence on intervention cost-effectiveness as basis for policy and implementation choices, as there are limitations on how relevant they are to what will happen in a particular country [18,19].

Because of the continued interest in scaling up health services, and the limited application of theoretical perspectives on scaling up in the current efforts to achieve the MDGs, it is a good time to examine what theoretical models have been used in scaling up for health in low and middle income countries. The purpose of this paper is to identify the theoretical frameworks that have been used to understand the issue of scaling up in the health sector in developing countries, and to identify how they inform practical questions of planning, implementation, monitoring, and evaluation for scale. It is hoped that by examining the theoretical approaches and lessons learned from them, we can gain insights for how to achieve the MDGs.

## Methods

We used a systematic approach to review the published and grey literature to identify and examine conceptual models for scaling up health programs in low and middle income countries. The areas of enquiry for understanding scaling up health services were identified through prior knowledge of the literature and discussions held with academics and professionals from development agencies (UNICEF, World Health Organization, GAVI Alliance, World Bank, Canadian International Development Agency, and United States Agency for International Development), and were intended to highlight the practical concerns about how to scale up. This resulted in the identification of a set of key domains and related questions, which are listed in Table 1. The key domains include the meaning or definition of scaling up used in the conceptual model, the type of resources required for scaling up, and the different perspectives on planning, implementation and evaluation. We also reviewed the political, social, and environmental context in which scaling up occurred, and the entity being scaled up (e.g. type of health service, health organization, or health program).

**Table 1:** Key Dimensions of Scaling Up Health Services

Scaling Up Dimension	Questions Addressed
Meaning of Scaling Up	How is scaling up defined? What is the time horizon for scaling up? How are valid strategies determined?

Scaling Up Resources	What types of resources are needed? How do we define absorptive capacity? What type of absorptive capacity is required for scaling up?

Planning Perspectives	How to plan for scaling up?

Implementation Perspectives	What are the key implementation components for scaling up?

Monitoring and Evaluation	How do we monitor and evaluate scaling up? Are the criteria for success aligned with the definition of scaling up?

To identify articles for review, we began with the World Health Organization (WHO) sponsored Expandnet database, with all 178 articles available on the site in March, 2009 subjected to initial review for relevance [20]. Expandnet, which began in 2003, is a global network of representatives from international organizations, non-governmental organizations, academic and research institutions, ministries of health and specific projects who seek to advance the science and practice of scaling up. Members of Expandnet include public health professionals who have been active participants in scaling-up initiatives including those implementing the WHO-sponsored Strategic Approach to Strengthening Reproductive Health Policies and Programmes in countries in Africa, Asia, Eastern Europe, and Latin America, individuals from technical assistance and donor agencies and people with expertise and interest in issues related to scaling up. An additional 22 articles were retrieved from a hand search of the references from these articles. We also used a systematic search of electronic databases using a set of relevant key words as search terms (see additional file 1), on studies published up to March 2009. The electronic resources included PubMed and websites for Human Resources for Health, the International Health Partnership, The WHO, The World Bank, GAVI, GFATM, the United States Agency for International Development, United Kingdom Department for International Development, PEPFAR, Commission on Macroeconomics and Health, JHPIEGO, Save the Children, Overseas Development Institute, United Nations Millennium Project, and Management Sciences for Health. This yielded another 72 articles for review. No year or language restrictions were placed on the electronic search. An article was eligible for review if it discussed the process of scaling up health services in a low and middle income country, as categorized by the World Bank [11]. To be included for review, the article needed to include a conceptual model for scaling up health services, which was defined as the identification of factors and their relationship to each other and the process of expanding health services, health programs, or other definition of scaling up in health. An article that described factors that influence MDG health outcomes, but did not define or describe a scaling up process, was not included in the review. After screening for duplicates and eligibility, 102 articles were included in the final review (listed in additional file 2). Data were abstracted from each article by one reviewer (SS) according to a standard set of questions concerning the conceptual models and the practical implications (Table 1), with further review by two other authors (JN and DHP) to arrive at a consensus in the case of uncertainty over the content. The results below are presented according to the domains of enquiry.

## Results

From the 102 articles reviewed, we identified six distinct conceptual models that focused on the question on how to scale up health services in developing countries, which are briefly summarized in Table 2[20-25]. Although three of the papers were published after 2000, none of the post-2000 frameworks were adopted by a global health initiative as a model for practice, nor were any developed specifically for scaling up services to achieve the MDGs. All frameworks tended to draw on lessons learned from scaling up experiences prior to the declaration of the MDGs. They were designed in the context of underlying concerns about how to expand and sustain experience gained from pilot projects or the diffusion of innovations in health, and so we have labeled these approaches broadly as "Scaling up Innovations and Pilot Projects". Although they differ in their emphases and content (e.g. some focus on scaling up of a health service, whereas others focus on the health technology or the organization providing services), they share similar perspectives about the meaning of scaling up, the resource requirements, and planning and implementation issues.

**Table 2:** Conceptual Frameworks Identified for Scaling Up Health Services

Name of Framework	Description of Framework	Year of Publication
A Learning Process Approach [21]	A model that describes a learning process to building program strategies and organizational competence. It suggests that a new program should progress through three developmental stages in which the focal concern is successively on learning to be effective, learning to be efficient, and learning to expand.	1980
Alternative Strategies for Scaling Up NGOs [22]	A model that describes four dimensions of scaling up of programs and organizations: (i) quantitative, (ii) functional, (iii) political and (iv) organizational development.	1995
Diffusion of Innovations [23]	Diffusion of innovations theory seeks to explain how, why, and at what rate new ideas and technology spread through cultures. The four main elements involve: (i) the innovation, (ii) communication channels, (iii) time and (iv) a social system	1995
SEED-Scale [24]	A model involving three principles for scaling up: (i) forming a three way partnership of community members, officials and experts, (ii) basing action on locally specific data, (iii) using a community work plan to change collective behavior	2002
Scaling Up Management (SUM) Framework [25]	A framework for those planning, implementing and funding pilot projects with the intention of scaling up. The three steps include: (i) developing a scaling up plan, (ii) establishing the pre-conditions for scaling up and (iii) implementing the scaling up process based on the identification of factors that can promote extension and sustainability	2003
Expandnet Framework [20]	A framework that presents the scaling up process within a systems context involving the following components: (i) determining the innovation, (ii) identifying the user organization, (iii) defining and analyzing the environment, (iv) identifying the resource team. It also involves identifies the need for considering the role of: (i) policy/legal/political scaling up, (ii) physical expansion of services and programs, (iii) diversification, and (iv) spontaneous scaling up	2008

### Meaning of scaling up

The health services being scaled up for the MDGs have involved innovations and pilot projects, though the strategies for scaling up have not been explicitly based on the theoretical understanding of innovations or how pilot projects can go to scale. The approaches used to "Scale up to Reach the MDGs" differ significantly from the theoretical approaches in their views on the dominant meaning of scaling up and the type of resources needed for scaling up health services, as well as their management approaches (Table 3). This table highlights these differences and forms the basis of our discussion comparing MDG strategies for scaling up to the strategies for scaling up innovations and pilot projects.

**Table 3:** Contrasting Meaning of Scaling Up, Resources Required, and Management Perspectives

*Dimension*	*Scaling up to Reach the MDGs*	*Scaling up Innovations and Pilot Projects*
**Meaning of scaling up**	"Becoming large": more people covered	"Expanding impact" and becoming sustainable in multiple dimensions, including quantitative, functional, organizational, and political terms
Time frame	Short to medium term	Medium to long term
Criteria for validity of scaling up strategies	Assumption of external validity of approaches. Search for easily replicable, standardized approaches	Assumption that approaches should be determined contextually. Internal validity of strategies being tested is most important. What works best depends on the particular context, time and place

**Scaling up Resources**	Money is a binding constraint; much money is needed	Money is necessary but not sufficient, and small amounts can go far. Money not usually the binding constraint.
Absorptive capacity	Ability to spend external funds	Ability to find a fit between: 1. Beneficiaries ability to voice concerns and how organizations that provide services make decisions 2. Requirements of programs and capabilities of organizations 3. Needs of beneficiaries and the resources and services made available

**Planning Perspectives**	Create better blueprints and targets that can be locally adapted	Learning by doing. Look for and embrace error, plan with key stakeholders, and link knowledge building with action

**Implementation Perspectives**	Range of well-defined managerial inputs, technologies, strategies and activities (often overlooking or assuming some constant quality) focused primarily on improved delivery of services	Mix of technocratic, political, social and economic activities and processes, which are not defined with specificity in advance. Service delivery outcomes alone is not the main outcome
	Focus on "accelerating" implementation to meet well-defined goals and deadlines	Slower, phased implementation, usually from the bottom up, which allows for systematic learning to emerge through incremental expansion based on concurrent, participatory research and adaptation
	Assumes that implementation will "occur", perhaps even spontaneous replication to new sites and beneficiaries once users see value of change	Acknowledges possibility of spontaneous replication, but strong bias towards "managed" implementation, including intensive monitoring and adaptation because of expected error and need for "champions", teamwork and capacity building

**Monitoring & Evaluation**	Focused on status of problem; uses formal surveys, rigor; written communication; statistical analysis; numerical presentation	Focused on problem-solving; uses observation, guided interviews, informant panels; timely feedback; oral communication; informed interpretation; narrative presentation

For large scale programs like GFTAM, GAVI, and PEPFAR, scaling up means increasing coverage of services through swift disbursement of funds, improving access, expanding partnerships, ensuring sustainable funding and promoting participatory ownership [3,26]. The specific definition for scaling up smaller innovations and pilot projects differs according to the goal of the framework, expectations of stakeholders, and outcomes of the processes. Yet these frameworks also tend to be more concerned than the MDG strategies with the process of scaling up, the adaptability of the innovation or service that is being scaled up, and the capacity of the organizations or communities that are implementing the expansion. For example, Uvin's framework identifies NGO expansion in four dimensions: 1. Quantitative: an organization increases size (including increasing human resources, financial resources and inputs) and coverage of people who are served; 2. Functional: an organization adds new activities or services to its existing work; 3. Political: an organization adds activities involving advocacy, empowerment, and making changes in policies; and 4. Organizational: an organization strengthens and adds variety to its financial sources and mechanisms and its organizational structures and functions [22]. The Expandnet framework defines success when there is an increase in the impact of health service innovations that have been successfully tested in pilot and experimental projects to foster policy and program development on a lasting basis [12].

Korten defines successful scaling up in three stages: 1. Adequate resources are provided along with technical input, capacity building, and understanding of community culture to demonstrate effectiveness; 2. Inputs per output are minimized along with assuring a good fit between the program requirements and the realistic capabilities of the organizations involved to demonstrate efficiency; and 3. Expansion through innovation and increasing organizational capacity to respond to large-scale requirements [21]. The Scaling up Management (SUM) framework developed by Kohl and Cooley builds on Korten's framework by identifying successful replication when other organizations increase their uptake and use of the innovation [25]. Successful collaboration occurs when formal and informal partnerships and networks are developed.

Taylor's SEED-Scale framework defines successful scaling up in terms of community involvement, where successful community projects are developed and promoted, and transformed into learning centers for other organizations seeking to learn how to implement the innovation, so that the projects are then systematically extended throughout different regions with other groups [24]. Rogers described his theories on the diffusion of innovation as focusing on the transfer of knowledge as the basis for successful scaling up of an innovation, with examples involving health behaviors or services [23].

### Absorptive Capacity

The ability to spend donor funds on activities related to the MDGs [27] and the macroeconomic implications of high volume of aid inflows are the primary absorptive capacity concerns of global health initiatives [28,29]. In contrast, Uvin focuses on overcoming human resource inadequacies at the operational level, developing efficient systems and policies for the smooth channeling of funds, and providing adequate incentives for efficient and effective use of resources to ensure that organizations have the adequate capacity to absorb and utilize funds for scaling up [22]. Absorptive capacity within the implementing organization has also been described as dependent upon the implementation capacity of the organization (adequate human resources, logistics and supplies, sound management, strong leadership, policy and legal framework set in place, supportive environment and adequate physical facilities) and the harmonization between the resource and user organization to ensure a smooth process of scaling up [12].

Diffusion of innovations theory stresses that the receptive context/climate of an organization is important to incorporate innovations and increase its absorptive capacity for new knowledge. Characteristics of a receptive context include presence of strong leadership; a clear strategic vision, both for the organization and for scaling up; good management relations; "champions" in critical positions; a climate that is conducive to experimentation and risk-taking; and effective monitoring systems to capture and use important data [30].

### Planning and Implementation Approaches

Although global health initiatives are concerned about promoting in-country participation and locally adapted goals, each has set up its own planning and proposal-writing process as well as parallel planning and review committees and processes. The GFTAM supports common funding mechanisms for joint funding in an effort to align Country Coordination Mechanisms (which are responsible for Global Fund proposals) with national planning mechanisms [31]. Various United States government entities, the host government, NGOs, and representatives from the corporate sector, multilateral institutions and other local stakeholders are all involved in PEPFAR planning, which is carried out in parallel with the national planning effort [3]. Planning for GAVI requires governments working with their interagency coordination committees and Health Sector Coordination Committees in preparing proposals for funding [4].

The approaches based on frameworks for innovations and scaling up pilot projects place less emphasis on initial planning, but stress the processes of learning by doing, embracing error, and linking knowledge-building with action as implementation is occurring. Kohl and Cooley provide a good example by breaking down the planning for scaling up into tasks involving problem identification, constituency building to support implementation, realigning resources and organizations, and ongoing performance monitoring and feedback [25].

### Implementation Perspectives

Much of the literature on implementation of large scale programs focuses on the need to overcome inadequate inputs to the system, including the lack of adequately trained and distributed staff, infrastructure, drugs and supplies; inadequate management and supervision systems; the lack of demand for health interventions or the physical, financial, or social barriers to care; and inadequate policies to promote the scaling up of health interventions [1]. This has led many agencies to focus on the technical aspects of improving implementation, as well as a renewed focus on strengthening the essential "building blocks" of health systems as a common basis for expanding coverage of health interventions [32-34].

In contrast, the framework by Uvin goes beyond constraints to implementing specific health services [22]. Packages of services are pilot tested and effectiveness is determined through local learning. Implementation is usually phased, often from the bottom up, allowing for incremental expansions based on participatory and concurrent research. This process also has a strong bias towards "facilitated" implementation rather than relying solely on spontaneous replication.

The SEED-Scale approach, which was grounded in past experiences of its investigators, was, in some ways, an attempt to provide an alternative approach to the campaigns that had dominated smallpox and polio eradication [24]. In China, a multi-phased pilot project was mounted in ten model counties, each of which focused on the major health problems of children to significantly reduce disease and death. The model counties were transformed into demonstration sites where experimental townships served as training sites for workers from other townships and counties. The scale-up process was rolled out incrementally; the numbers of counties and expenditures for each phase were gradually increased, across the country. Several enabling factors for the scaling up process were identified: 1. Action training, which was experience-based and participatory with continuous evaluation; 2. Priority setting, in which key health interventions for each region focused on developing cost-effective solutions; and 3. Exponential extension where capacity and training of health education staff was upgraded.

In the models for reaching the MDGs, monitoring and evaluation is viewed as measuring final results in terms of project outputs, coverage and projected health impacts with little focus on the challenges and successes of the model itself. In the models of scaling up innovations and pilot projects, evaluation tends to emphasize the learning to be achieved from monitoring and the involvement of key stakeholders (often involving beneficiaries, implementers, sponsors, and experts). The need for feedback mechanisms to provide transparency to all players, and not just managers, on the successes and challenges in scaling up a particular strategy is seen as important. These approaches also tend to measure the processes of scaling up. The SUM framework stresses the importance of monitoring to track the effects of changes from pilot projects or initial scale up results (i.e. intermediate outcomes/process indicators) to make the necessary adjustments as the project scales up further [25].

## Discussion

Although this paper does not capture all the subtleties of the different global health initiatives or models for scaling up, it is clear that the current focus on quantitative coverage targets provides little insight for the actions needed for further growth or sustainability. Rather, the lessons from the past suggest that attention is needed to political, organizational, and functional dimensions of scaling up, and the need to nurture local organizations. The current bias toward supply side solutions and over-reliance on the public sector also ignores the importance of demand side factors and influence of market systems that are often critical for long-term success [35]. Most global health initiatives largely fund public sector programs, often through budget support to governments that rely on government systems, or by creating special projects that bypass usual government systems, sometimes relying on large NGOs [19].

Each of the scaling up models described here also challenge the utility of any "one size fits all" strategy or the assumption that strategies can simply be adapted to local conditions. Rather, they suggest that specific strategies need to be developed in ways that are appropriate to individual countries and areas within a country, so that they can better involve and build local institutions and address local contexts. Systematic reviews of empiric research on strengthening health services support this view, and have demonstrated that no single strategy or blueprint has been shown to repeatedly strengthen health services in developing countries [19]. But successful processes to improve service delivery have been identified, including: stakeholder consultation and involvement; explicitly identifying and minimizing constraints to implementation; feedback mechanisms that allow continuous adaptation to local dynamic contexts; and seeking larger effects through multiple rather than single component strategies [19]. Blueprint approaches are rarely adaptive enough to work in predictable ways in different contexts, and are likely to produce unintended consequences, which can lead to poorly functioning and unsustainable interventions. By the same token, local adaptations are unlikely to be easily scaled up on their own; changing the institutional arrangements for service providers, however, can strongly influence their performance and prospects for scaling up. Yet approaches that involve partnerships among communities, policy makers, and experts have been more successful than approaches that do not create such partnerships [19].

Promoting participatory approaches for implementation and local autonomy does not guarantee success, but it appears to be a critical factor contributing to ownership and sustainability of programs. But rigid models for standardized implementation that exclude national and local institutions in matters of design, resource allocation, and monitoring, particularly those outside of individual projects or programs, ensure that local institutions will remain disempowered and peripheral to the scaling up process.

It is not clear whether the global health initiatives will be able to pursue any radically different approaches to scaling up. To date, the resources provided for scaling up health services to reach the MDGs are larger than those available in the past to scaling up innovations and pilot projects. The increased political and financial attention have created new opportunities for expanding access to essential health services around the world, and has brought a much needed sense of urgency. Whereas it is clearly desirable to have quick action and results, the current approaches show that this cannot be accomplished hastily. Learning lessons from experience and models for change will provide new opportunities to tackle the complexities of scaling up in locally relevant and accountable ways.

## Conclusions

Although there is agreement on the importance of scaling up health services to achieve the MDGs, the dominant paradigm for scaling up pursued by global health initiatives has focused excessively on providing adequate funding and rapidly expanding the coverage of health services in a mechanistic way. Such an approach is overly simplistic and not consistent with the experience that each country follows its own pathway in changing health services coverage and health outcomes. The current emphasis on achieving health services targets provides little insight on how to build the organizational, functional, and political capabilities that are needed for scaling up. We examined alternative models for scaling up, which have applied theories of change that demonstrate how scaling up occurs in complex and dynamic environments. Experience with these approaches suggest that rather than relying on blueprint planning and costing that is prevalent among global health initiatives, a "learning by doing" approach that engages key stakeholders, uses data to address constraints, and incorporates results from pilot projects, is a more promising approach to finding pathways to scaling up. These lessons should be applied to current strategies to achieve the MDGs.

## Abbreviations

GAVI: Global Alliance for Vaccines Initiative; GFATM: Global Fund to Fight AIDS, Tuberculosis, and Malaria; HIV/AIDS: Human Immunodeficiency Virus/Acquired Immune Deficiency Syndrome; MAP: Multi-Country HIV/AID Program (World Bank); MDGs: Millennium Development Goals; NGOs: Non-governmental organizations; PEPFAR: US President's Emergency Fund for AIDS Relief; SEED (in SEED-Scale): Self-Evaluation for Effective Decision-making; SUM: Scaling Up Management; WHO: World Health Organization.

## Competing interests

The authors declare that they have no competing interests.

## Authors' contributions

DHP, JN, and SS conceptualized the paper, and designed the literature review. SS participated in the design of the study and carried out the literature review. JN participated in the conceptualization and design of the study and the analysis of results. DHP participated in the conceptualization and design of the study. TM undertook the analysis of trends in under five mortality. All authors contributed to drafting the manuscript and have read and approved the final manuscript.

## Pre-publication history

The pre-publication history for this paper can be accessed here:

http://www.biomedcentral.com/1472-6963/11/336/prepub

## Supplementary Material

Additional File 1 **Key Word Search Terms**. Search terms used to identify articles for review. Click here for file

Additional File 2 **Scaling Up Articles Reviewed**. List of articles reviewed concerning frameworks for scaling up in health. Click here for file
