# Supplementary material for: Do we have the right models for scaling up health services to achieve the Millennium Development Goals?
Source: BMC Health Serv Res. 2011 Dec 14;11:336. doi: 10.1186/1472-6963-11-336 (PMC3260120; doi:10.1186/1472-6963-11-336)
Supplement: Additional File 1 — Key Word Search Terms. Search terms used to identify articles for review. [file 1472-6963-11-336-S1.DOC]

**Do We Have the Right Models for Scaling Up Health Services to Achieve the Millennium Development Goals?**

**Additional File 1**

**Key Word Search Terms:**

scaling up, MDGs, human resources, health systems, frameworks, implementation, PEPFAR, GAVI, GFTAM, HIV/AIDS, coverage, immunization, community participation, financial resources, absorptive capacity, innovations, pilot projects, implementation, planning, definition, monitoring.
