# Supplementary material for: Do we have the right models for scaling up health services to achieve the Millennium Development Goals?
Source: BMC Health Serv Res. 2011 Dec 14;11:336. doi: 10.1186/1472-6963-11-336 (PMC3260120; doi:10.1186/1472-6963-11-336)
Supplement: Additional File 2 — Scaling Up Articles Reviewed. List of articles reviewed concerning frameworks for scaling up in health. [file 1472-6963-11-336-S2.DOC]

**Do We Have the Right Models for Scaling Up Health Services to Achieve the Millennium Development Goals?**

**Additional File 2 Scaling Up Articles Reviewed**

**List of articles reviewed concerning frameworks for scaling up in health**

1. Advance Africa. 2001. “Scaling-up Family Planning and Reproductive Health Programs: Resources and Publications.” [accessed on October 10, 2010]. Available at: <http://www.advanceafrica.org/>
2. Askew, I., and H. Evelia. 2007. “Mainstreaming and Scaling up the Kenya Adolescent Reproductive Health Project.” Frontiers Project, Population Council. Nairobi, Kenya
3. Attawell, K. 2004. “Going to Scale in Ethiopia: Mobilizing Youth Participation in a National HIV/AIDS Program.” Social & Scientific Systems, Inc./The Synergy Project. Washington DC
4. Bass, E. 2005. “The two sides of PEPFAR.” The Lancet 365 (9477):2077-2078
5. Bajpai, N., and R.H. Dholakia. 2006. “Scaling Up Primary Health Services in Rural Rajasthan: Public Investment Requirements and Policy Reform." CGSD Working Paper No. 32. Center on Globalization and Sustainable Development, The Earth Institute at Columbia University
6. Bennett, S., J.T. Boerma, and R. Brugha. 2006. “Scaling up HIV/AIDS evaluation.” The Lancet 367 (9504): 79-82
7. Binswanger, H.P., and S.S. Aiyar. 2003. “Scaling Up Community Driven Development Theoretical Underpinnings and Program Design Implications.” The World Bank. Washington DC
8. Billings, D.L., B.B. Crane, J. Benson, J. Solo, and T. Fetters. 2007. “Scaling-up a public health intervention: A comparative study of post-abortion care in Bolivia and Mexico.” Social Science and Medicine 64: 2210-2222
9. Bloom, G., C. Champion, H. Lucas, M.H. Rahman, A. Bhuiya, O. Oladimeji, and D.H. Peters. 2008 “Health markets and future health systems: Innovations for equity.” Global Forum Update on Research for Health 5: 30-33.
10. Bloom, G., H. Standing, and A. Joshi. 2009. “Institutional Context of Health Services.” In *Improving Health Service Delivery in Developing Countries.* Edited by D.H. Peters, S. El-Saharty, B. Siadat, K. Janovsky, M.Vujicic, pp. 203-213. Washington D.C: World Bank
11. Brugha, R., M. Starling, and G. Walt. 2002. “GAVI, the first steps: lessons for the Global Fund.” The Lancet 359 (9304): 435-438
12. Chambers, R. 1992. “Spreading and self-improving: A strategy for scaling-up.” In *Making a difference: NGOs and Development in a Changing World.* Edited by M. Edwards and D. Hulme. London, England: Save the Children/Earthscan
13. Chopra, M, and N. For.. 2005. “Scaling up health promotion interventions in the era of HIV/AIDS: challenges for a rights based approach.” Health Promotion International 20 (4)
14. Core Group. 2005. “Scale and Scaling-Up: A CORE Group Background Paper on Scaling-Up Maternal, Newborn and Child Health Services.” Washington, D.C.
15. Countdown to 2015 Core Group. 2008. “Countdown to 2015 for maternal, newborn, and child survival: the 2008 report on tracking coverage of interventions.” The Lancet 371:1247-58
16. DeJong, J. 2001. “A question of scale? The challenge of expanding the impact of non-governmental organizations’ HIV/AIDS efforts in developing countries.” Washington, DC: Horizons Project, Population Council
17. DeJong, J. 2003. “Making an Impact in HIV and AIDS: NGO Experiences of Scaling Up.” London, England: Intermediate Technology Development Group Publishing.
18. De Renzio, P. 2005. “Scaling up versus absorptive capacity: challenges and opportunities for reaching the MDGs in Africa.” ODI Briefing Paper. London: Overseas Development Institute
19. De Souza, R.M. 2008. "Scaling up Integrated Population, Health and Environment Approaches in the Philippines: A review of early experiences." Washington, DC: World Wildlife Fund and the Population Reference Bureau
20. Department for International Development. 2002. “The Macroeconomic Effects of Aid.” London: Department for International Development
21. Farr, C., and A. Natalie. 2008. “Using Diffusion of Innovation Theory to Encourage the Development of a Children’s Health Collaborative: A Formative Evaluation.” Journal of Health Communication 13 (4): 375-388
22. Garrett, L. 2007. “The challenge of global health.” Foreign Affairs 86: 1-17
23. GAVI, 2008. “GAVI Alliance Handbook: Country Proposal and Monitoring Processes.” Geneva, Switzerland
24. Gillespie, D., S. Karklins, A. Creanga, S. Khan, and N. Cho. 2007. “Scaling Up Health Technologies.” Baltimore, MD: Johns Hopkins University and Bill & Melinda Gates Foundation
25. Gillespie, S. 2004. “Scaling Up Community-Driven Development: A Synthesis of Experience.” Washington, D.C.: International Food Policy Research Institute
26. Gladwell, M. 2000. The Tipping Point: How Little Things Can Make a Big Difference. Boston, MA: Little, Brown and Company.
27. Glaser, E.M. 1983. “**Putting Knowledge to Use: Facilitating the Diffusion of Knowledge and the Implementation of Planned Change.”** San Francisco, California: Jossey-Bass Inc.
28. Gottret, P, and G. Schieber. 2006. Health Financing Revisited: A Practitioner’s Guide. Washington, D.C.: World Bank.
29. Greenhalgh, T., G. Robert, F. Macfarlane, P. Bate, and , O. Kyriakidou. 2004. “Diffusion of Innovations in Service Organizations: Systematic Review and Recommendations.” The Milbank Quarterly 82 (4): 581-629.
30. Hanson, K., S. Cleary, H. Schneider, S. Tantivess, and L. Gilson. 2010. “Scaling up health policies and services in low-and middle-income settings.” BMC Health Services Research 10 (Suppl 1): I1
31. Hanson, K., M.K. Ranson, V. Oliveira-Cruz, and A. Mills. 2003. “Expanding access to priority health interventions: a framework for understanding the constraints to scaling-up.” Journal of International Development 15:1-14.
32. Hardon, A., and S. Blume. 2005. “Shifts in global immunization goals (1984-2004): unfinished agendas and mixed results.” Social Science and Medicine, 60: 345-356
33. Helfenbein, S., and C.A. Severo. 2004. “Scaling up HIV/AIDS Programs: A manual for multi-sectoral planning.” Washington, *D.C.:* Management Sciences for Health
34. High-Level Forum on the Health MDGs 2004. “MDG-oriented Sector and Poverty Reduction Strategies: Lessons from Experience in Health.” *Proceedings from the High-Level Forum on the Health MDGs* in Abuja, Nigeria
35. Howard-Grabman, L., and G. Snetro. 2006 “ How to Mobilize Communities for Health and Social Change - Chapter Seven - Prepare to Scale Up.” Washington, D.C. Save the Children
36. Howes, M., and M.G. Sattar. 1992. “Bigger and better? Scaling-up strategies pursued by BRAC 1972-1991.” In *Making a difference: NGOs and Development in a Changing World*. Edited by Edwards M, Hulme D. London, England: Save the Children/Earthscan
37. Huicho, L., M. Davila, M. Campos, C. Drasbek, J. Bryce, and C.G. Victora. 2005. “Scaling up Integrated Management of Childhood Illness to the national level: achievements and challenges in Peru.” Health Policy and Planning 20: 14-24.
38. International Monetary Fund. 2007. “Fiscal Policy Response to Scaled-up Aid.” Washington, DC: International Monetary Fund
39. Johns, B., and R. Baltussen. 2004. “Accounting for the cost of scaling-up health interventions.” Health Economics 13: 1117-1124
40. Johns, B., and T. Tan Torres. 2005. “Costs of scaling up health interventions: a systematic review.” Health Policy Plan 20: 1-3.
41. Joint Learning Initiative. 2004. “Human Resources for Health: Overcoming the Crisis.” Washington, DC: Global Health Initiative, Harvard University
42. Knippenberg, R., J.E. Lawn, G.L. Darmstadt, G. Begkoyian, H. Fogstad, N. Walelign, and V.K. Paul. 2005. “Neonatal Survival 3: Systematic Scaling Up of Neonatal are in Countries.” The Lancet 365 (9464): 1087.
43. Kohl, R, and L. Cooley. 2003. “Scaling Up-A Conceptual and Operational Framework.” Washington, D.C.: Management Systems International
44. Kohl, R, and L. Cooley. 2005. “Scaling Up-From Vision to Large Scale Change: A management framework for practitioners.” Washington DC: Management Systems International
45. Korten, D.C. 1980. “Community Organization and Rural Development: A Learning Process Approach.” Public Administration Review 40 (5): 480-511.
46. Korten, D.C., and R. Klauss. 1984. People Centered Development*.* Kumarian Press. West Hartford Connecticut
47. Kurowski, C., K. Wyss, S. Abdulla, and A. Mills. 2007. “Scaling up Priority health interventions in Tanzania: the human resources challenge.” Health Policy and Planning 22 (3): 113-127
48. Lane, C., and A. Glassman. 2007. “Bigger and Better: Scaling Up And Innovation in Health Aid.” Health Affairs 26 (4): 935-948
49. Levine, R., ‘What Works’ Working Group, Kinder, M. 2004. Millions Saved: Proven Successes in Global Health. Washington, DC: Center for Global Development
50. Magesa, S.M., C. Lengeler, D. deSavigny, J.E. Miller, R.J.A. Njay, K. Kramer, A. Kitua, and A. Mwita. 2005. “Creating an “enabling environment” for taking insecticide treated nets to national scale: The Tanzanian experience” Malaria Journal 4 (34)
51. Management Sciences for Health (MSH). 2007. "Implementing Best Practices in Reproductive Health." Cambridge, MA: USAID & WHO
52. Management Sciences for Health (MSH). 2002. “Ten dimensions of scaling up reproductive health programs: an introduction.” USA Agency for International Development (USAID)
53. Management Sciences for Health (MSH). 2007. “A guide for fostering change to scale up effective health services.” Implementing Best Practices Consortium.
54. Mangham, L.J., and K. Hanson. 2010. “Scaling up in international health: what are the key issues?” Health Policy Planning 25: 85-96
55. Matsubayashi, T., D.H. Peters, M.H. Rahman. 2009. “Analysis of Cross-Country Changes in Health Services.” In *Improving Health Service Delivery in Developing Countries.* Edited by D.H. Peters, S. El-Saharty, B. Siadat, K. Janovsky, M.Vujicic, pp. 173-202. Washington D.C: World Bank
56. McCannon, C.J., D.M. Berwick, and M.R. Massoud. 2007. “The Science of Large-Scale Change in Global Health.” Journal of the American Medical Association 298:1937-1939.
57. Moore, A., and J.S. Morrison*.* 2007. “Health Worker Shortages Challenge PEPFAR Options for Strengthening Health Systems.” Center for Strategic and International Studies (CSIS)
58. International Monitoring Fund 2005. “Review of the Poverty Reduction Strategy Approach: Balancing Accountabilities and Scaling Up Results.” Washington D.C.: International Monitoring Fund.
59. MDG Africa Steering Group. 2008. “Achieving the Millennium Development Goals in Africa: Recommendations of the MDG Africa Steering Group.” New York: The United Nations
60. Medlin, C.A., M. Chowdhury, D.T. Jamison, and A.R. Measham. 2006. “Improving the health of populations: lessons of experience.” In *Disease control priorities in developing countries.* 2nd edition. Edited by D.T. Jamison, J.G. Berman, A.R. Measham, G. Alleyne, M. Claeson, B.D. Evans and P. Musgrove, pp.181-194. Washington, D.C: World Bank
61. Nsutebu, E.F., J.D. Walley, E. Mataka, C.F. Simon. 2001. “Scaling-up HIV/AIDS and TB home-based care: lessons from Zambia.” Health Policy and Planning 16(3): 240-247.
62. Nyonator, F.K., J.K. Awoonor-Williams, P.F. Phillips, T.C. Jones, and R.A. Miller. 2005. "The Ghana Community-based Health Planning and Services Initiative for scaling up service delivery information." Health Policy and Planning 20 (1): 25-34
63. Overseas Development Institute. 2005. “Incentives for Harmonization in Aid Agencies.” ODI. London, England
64. Ovretveit, J., B. Siadat, D.H. Peters, A. Thota, and S. El-Saharty. 2009. “ Review of Strategies to Strengthen Health Services.” In *Improving Health Service Delivery in Developing Countries.* Edited by D.H. Peters, S. El-Saharty, B. Siadat, K. Janovsky, M.Vujicic, pp. 35-54 Washington D.C: World Bank
65. “Paris Declaration on Aid Effectiveness.” 2005. [accessed on October 10, 2010]. Available at <http://www.aidharmonization.org/ah-overview/secondary-pages/editable?key=205>
66. Partners for Health ReformPlus. 2004. “The role of pilot programs: Approaches to health systems strengthening.” Bethesda, MD: PHRPlus, Abt Associates.
67. PEPFAR. 2004. “U.S. Five-Year Global HIV/AIDS Strategy.” Washington, D.C.
68. Peters, D.H., S. El-Saharty, B. Siadat, K. Janovsky, M. Vujicic. (Eds.). 2009. Improving Health Service Delivery in Developing Countries Washington D.C: World Bank
69. PRSP as a Framework for Scaling up Efforts to reach the MDGs 2006. *Symposium conducted at the Africa Conference of African Ministers of Finance* from 21-22 May 2006, Abuja, Nigeria.
70. Pokhrel, S. 2006. “Scaling up health interventions in resource-poor countries: what role does research in stated-preference framework play?” Health Research Policy and Systems 4:4.
71. Rassekh, B., and N. Segaran. 2009. “Review of Community Empowerment Strategies for Health.” In *Improving Health Service Delivery in Developing Countries.* Edited by D.H. Peters, S. El-Saharty, B. Siadat, K. Janovsky, M.Vujicic, pp.127-142. Washington D.C: World Bank
72. Ravishankar, N., P. Gubbins, R.J. Cooley, K. Leach-Kemon, M.C. Michaud, D.T. Jamison, C.L.J. Murray. 2009. “Financing of global health: tracking development assistance for health from 1990 to 2007.” Lancet 373: 2113-24.
73. Robb-McCord, J., and W. Voet. 2003. “Scaling up practices, tools, and approaches in the maternal and neonatal health program.” Baltimore, MD: JHPIEGO Corp.
74. Rogers, M.E. 1995. Diffusion Of Innovations: Fourth Edition. New York: The Free Press
75. Snetro-Plewman, G., M. Tapia, V. Uccellani, A. Brasington, and M. McNulty. 2007. “Taking Community Empowerment To Scale: Lessons from Three Successful Experiences.” Baltimore, MD: Health Communication Partnership, Johns Hopkins Bloomberg School of Public Health.
76. Subramanian, S., and D.H. Peters. 2009. “Evaluation of Changes in Health Results in World Bank-Assisted Health Projects.” In *Improving Health Service Delivery in Developing Countries.* Edited by D.H. Peters, S. El-Saharty, B. Siadat, K. Janovsky, M.Vujicic, pp. 217-227. Washington D.C: World Bank
77. Schneider, H., D. Coetzee, D. Van Rensburg, and L. Gilson. 2010. “Differences in antiretroviral scale up in three South African provinces: the role of implementation management.” BMC Health Services Research 10 (Suppl 1): S4
78. Simmons, R., P. Fajans, and L. Ghiron. (Eds.). 2008. Scaling up Health Service Delivery: From Pilot Innovations to Policies and Programmes. Geneva, Switzerland: World Health Organization
79. Simmons, R., J. Brown, and M. Diaz. 2002. “Facilitating Large-scale Transitions to Quality of Care: An Idea Whose Time Has Come.” Studies in Family Planning 33 (1): 61-75
80. Stenberg, K., B. Johns, R.W. Scherpbier, T. Tan-Torres, and T. Edejer. 2007. “A financial road map to scaling up essential child health interventions in 75 countries.” Bulletin of the World Health Organization, 85(4): 305-316
81. Sternin, M., J. Sternin, and D. Marsh. 1999. “Scaling up poverty alleviation and nutrition program in Vietnam.” In *Scaling Up, Scaling Down - Overcoming Malnutrition in Developing Countries*. Edited by T.J. Marchione. Australia: Gordon and Breach
82. Taylor-Ide, D., and E.C. Taylor, 2002. Just and Lasting Change: When Communities Own Their Futures*.* Baltimore, MD: The Johns Hopkins University Press
83. The Global Fund. 2007. “The Global Fund Annual Report 2007.” Geneva, Switzerland
84. The Global Fund. 2008. “A Report on the Country Coordinating Mechanism Model: Lessons Learned in the field. Health Financing and Governance.” Geneva, Switzerland
85. The World Bank. 2004. “Poverty Strategy Papers-Progress in Implementation.” Washington, DC: The World Bank
86. The World Bank. 2008. “World Development Indicators CD-ROM 2008.” Washington, DC: The World Bank
87. The World Bank. 2007. Healthy Development: The World Bank Strategy for Health, Nutrition, and Population Results. Washington, DC: World Bank and Oxford University Press
88. Travis, P., S. Bennett, A. Haines, T. Pang, Z. Bhutta, N.A. Hyder, R.N. Pielemeier, A. Mills, and T. Evans. 2004. “Overcoming healthy-systems constraints to achieve the Millennium Development Goals.” The Lancet, 364: 900-906
89. United Nations. 2008. “The Millennium Development Goals Report 2008.” New York
90. UN Millennium Project. 2005. “Investing in Development. A Practical Plan to Achieve the Millennium Development Goals.” UN Millennium Project. New York
91. USAID 2001. “Expanding and strengthening community action: A study to explore ways to scale up effective, sustainable community mobilization interventions to mitigate the impact of HIV/AIDS on children and families.” Displaced Children and Orphans Fund of USAID Washington DC
92. Uvin, P. 1995. “Fighting hunger at the grassroots: Paths to scaling up.” World Development 23 (6): 927-939.
93. Uvin, P., P.S. Jain, and L.D. Brown. 2000. “Think Large and Act Small: Toward a New Paradigm for NGO Scale Up.” World Development 28 (8): 1409-1419
94. Uvin, P., and D. Miller. 1996. “Paths to scaling-up: Alternative strategies for local nongovernmental organizations.” Human Organization 55: 344-354
95. Wagstaff, A., M. Claeson, R.M. Hecht, P. Gottret, and Q. Fang. 2006. "Millennium Development Goals for Health: What Will It Take to Accelerate Progress?" In *Disease control priorities in developing countries.* 2nd edition. Edited by D.T. Jamison, J.G. Berman, A.R. Measham, G. Alleyne, M. Claeson, B.D. Evans and P. Musgrove, pp.181-194 Washington, D.C: World Bank
96. The World Bank, 2004. “World Development Report: Making Services Work for Poor People.” Washington, DC: World Bank and Oxford University Press
97. The World Health Organization 2007. “Scaling Up Health in Cambodia.” Geneva, Switzerland
98. World Health Organization 2006. “World Health Report 2006: Working Together for Health.” Geneva, Switzerland
99. World Health Organization 2007. “Everybody’s Business: Strengthening Health Systems to Improve Health Outcomes: WHO’s Framework for Action.” Geneva, Switzerland
100. WHO Commission on Macroeconomics and Health, 2001. “Macroeconomics and Health: Investing in Health for Economic Development. Report of the Commission on Macroeconomics and Health.” Geneva, Switzerland: World Health Organization
101. WHO Commission on Macroeconomics and Health, 2001. “Constraints to Scaling Up Health Interventions: Country Case Study: Chad.” Geneva, Switzerland: World Health Organization
102. Yothasamut, J., C. Putchon, T. Sirisamutr, Y. Teerawattananon, and S. Tantivess. 2010. “Scaling up cervical cancer screening in the midst of human papillomavirus vaccination advocacy in Thailand.” BMC Health Services Research 10 (Suppl 1): S5.
